# GATCDA: Predicting circRNA-Disease Associations Based on Graph Attention Network

**DOI:** 10.3390/cancers13112595

**Published:** 2021-05-25

**Authors:** Chen Bian, Xiu-Juan Lei, Fang-Xiang Wu

**Affiliations:** 1 School of Computer Science, Shaanxi Normal University, Xi’an 710119, China; bianchen@snnu.edu.cn; 2 Division of Biomedical Engineering, University of Saskatchewan, Saskatoon, SK S7N 5A9, Canada

**Keywords:** circRNA–disease association, graph attention network, circRNA–miRNA–mRNA axis

## Abstract

**Simple Summary:**

CircRNAs (circular RNAs), a novel kind of non-coding RNAs, play a regulatory role in cellular processes. A growing number of biological experiments has proved that circRNAs can be used as biomarkers and therapeutic targets of some cancers. As the time and financial costs of biological experiments are high, computational methods have become a better way to predict the associations between circRNAs and diseases. Graph attention network was first applied to predict circRNA-disease associations with multiple similarities of data in this study. The circRNA–miRNA interactions and disease-mRNA interactions were adopted to construct features. The computational method proposed in this study has improved the prediction performance.

**Abstract:**

CircRNAs (circular RNAs) are a class of non-coding RNA molecules with a closed circular structure. CircRNAs are closely related to the occurrence and development of diseases. Due to the time-consuming nature of biological experiments, computational methods have become a better way to predict the interactions between circRNAs and diseases. In this study, we developed a novel computational method called GATCDA utilizing a graph attention network (GAT) to predict circRNA–disease associations with disease symptom similarity, network similarity, and information entropy similarity for both circRNAs and diseases. GAT learns representations for nodes on a graph by an attention mechanism, which assigns different weights to different nodes in a neighborhood. Considering that the circRNA–miRNA–mRNA axis plays an important role in the generation and development of diseases, circRNA–miRNA interactions and disease–mRNA interactions were adopted to construct features, in which mRNAs were related to 88% of miRNAs. As demonstrated by five-fold cross-validation, GATCDA yielded an AUC value of 0.9011. In addition, case studies showed that GATCDA can predict unknown circRNA–disease associations. In conclusion, GATCDA is a useful method for exploring associations between circRNAs and diseases.

## 1. Introduction

CircRNAs (circular RNAs) are a class of non-coding RNA molecules with a closed circular structure, without a 5′-end cap and a 3′-end ployA tail. They are mainly located in the cytoplasm or stored in exosomes, and are not affected by RNA exonuclease [1]. Although circRNAs are non-coding RNAs, some circRNAs can encode polypeptides. Currently, biological functions of circRNAs are well-recognized as follows [2]: miRNA sponges, regulatory protein binding, regulation of gene transcription, and coding functions. CircRNA expression is more stable and not easily degradable, and has been proved to exist widely in a variety of eukaryotes [1]. Most circRNAs are formed by exon loops, and some circRNAs are lariat structures formed by intron loops. Because circRNAs contain a number of miRNA response elements (MREs), they can form the catalytic core of the RNA-induced silencing complex (RISC) with AGO proteins, which eventually leads to the degradation of circRNAs [3]. According to their sources, circRNAs can be roughly divided into four categories [4]: full-exon circRNAs, exon-introns circRNA (EIcircRNAs), intron-composed lariat circRNAs, and circRNAs produced by cyclization of viral RNA genomes (tRNA, rRNA, snRNA, etc.). Twenty years ago, scientists found circRNAs from plant viroids, yeast mitochondria, and hepatitis B viruses (HBV) as byproducts of abnormal splicing that have no regulatory function [5]. In 2013, Hansen et al. proposed and confirmed for the first time that circRNA is the regulatory mechanism of the miRNA sponge [6], providing a new field for circRNA research. With the rapid development in RNA sequencing technology and bioinformatics analysis, 14,807 candidate circRNAs have been identified in the human tissue transcriptome, and many exons have been found to form circRNAs by nonlinear reverse splicing or gene rearrangement in cells of other species. 

In recent years, many studies [7,8,9] have shown that circRNAs are closely related to the occurrence and development of diseases, and predicted circRNAs’ application prospects in aspects of diagnostic markers of diseases. For example, after brain/spinal cord injury, circRNAs can activate several biological, molecular, and cellular activities. Therefore, interventions centered on the regulation of circRNAs may be promising for traumatic brain injury and spinal cord injury [10]. Chen et al. demonstrated that circRNA circCTNNA1 promoted colorectal cancer progression by sponging miR-149-5p and regulating FOXM1 expression [11]. Wang et al. found that circCNST promoted the tumorigenesis of osteosarcoma cells by sponging miR-421 and targeting SLC25A3, providing a potential biomarker for patients with osteosarcoma [12]. Wu et al. discovered that circ_0009582, circ_0037120, and circ_0140117 may serve as potential biomarkers for predicting the occurrence of hepatocellular carcinoma in patients with HBV infection [13]. Li et al. revealed that circRNA circITGA7 may play a regulatory role in thyroid cancer and may be a potential marker for thyroid cancer diagnosis or progression [14].

At present, the number of known interactions between circRNAs and diseases obtained through biological experiments is increasing. Some relevant databases have appeared [15,16,17,18], including the interactions between circRNAs and diseases verified by biological experiments. Due to the significant time and financial costs associated with biological experiments, in recent years, it has become a hot topic to predict associations between circRNAs and diseases using computational methods with these databases. The current calculation methods can be divided into five categories [19]. The first category is a network propagating method. For example, the computational model BRWSP applies biased random walk to search paths on a multiple heterogeneous network to discover circRNA-disease associations [20]. A method called KATZHCDA uses KATZ measures for human circRNA-disease association prediction [21]. The second category is a recommendation system method. For example, Lei et al. proposed a computational method named ICFCDA based on collaboration filtering recommendation system, handling the “cold start” problem to predict potential circRNA-disease associations [22]. The third category is the matrix completion methods. For example, a computational method called iCircDA-MF was developed based on matrix factorization by Wei et al. [23]. Zhang et al. [24] utilized metapath2vec++ and matrix factorization to discover circRNA-disease associations. The fourth category is the classical machine learning methods. For example, based on a gradient boosting decision tree, a model named GBDTCDA was proposed by Lei et al. in 2019 [25]. A computational model called RWLR uses logistic regression to predict circRNA-disease associations [26]. The fifth category is the deep learning methods. For example, Wang’s method [27] applies a convolutional neural network to discover unknown circRNA-disease associations. In 2020, GCNCDA was proposed based on a graph convolutional network [28]. 

In this paper, we propose a novel computational model named GATCDA to predict circRNA-disease associations with graph attention network (GAT). First, we construct a circRNA-disease association network, a circRNA-miRNA association network and a disease-mRNA association network. Second, we calculate disease symptom similarity, network similarity and information entropy similarity for both circRNAs and diseases. Third, these similarities are integrated to create the features of circRNAs and diseases. Fourth, the circRNA-disease association network and the features of circRNAs and diseases are fed into GAT, and the output is the prediction score of associations between circRNAs and diseases.

## 2. Materials and Methods

**Figure 1:**
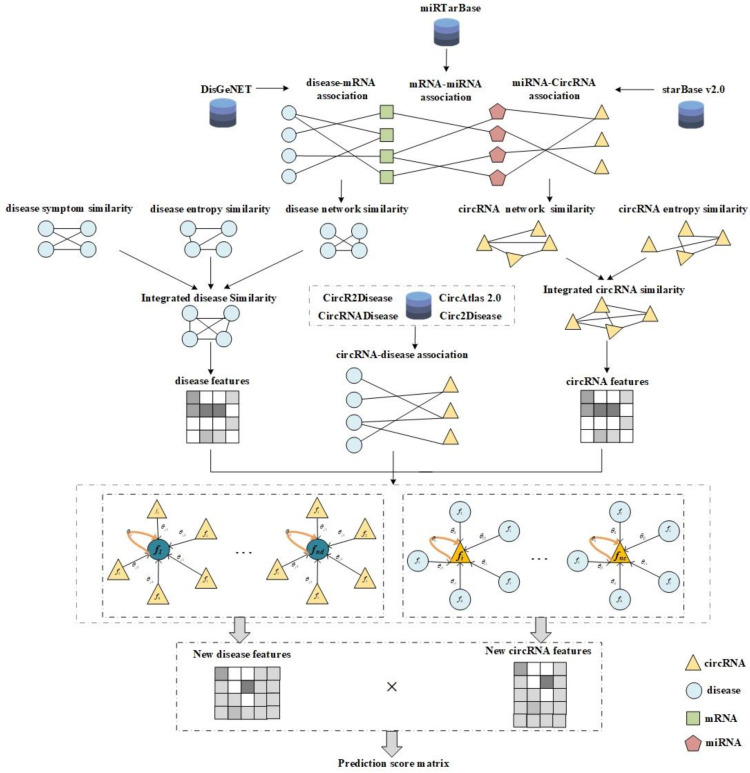
The flowchart of the computational method GATCDA.

The whole flowchart of GATCDA is shown in Figure 1.

### 2.1. Dataset Curation

#### 2.1.1. CircRNA-Disease Association

The circRNA-disease associations were downloaded from CircR2Disease [15], CircAtlas 2.0 [16], Circ2Disease [17], and CircRNADisease [18], in which the number of circRNA-disease associations are 739, 927, 273, and 354, respectively. After integration, we obtained 768 circRNA-disease associations, including 624 circRNAs and 102 diseases.

#### 2.1.2. CircRNA-MiRNA Association and Disease-mRNA Association

An initial circRNA-miRNA association dataset was downloaded from the starBase v2.0 [29] including 130,000+ circRNA-miRNA interactions, with 276 miRNA entries and 7018 circRNA entries. We selected 82 circRNAs common to circRNA-disease interactions and circRNA-miRNA interactions. There were only 142 miRNAs related to these 82 circRNAs in the initial circRNA-miRNA interactions. Finally, we constructed a new circRNA-miRNA association network including 509 circRNA-miRNA associations among 142 miRNAs and 82 circRNAs.

An initial disease-mRNA association dataset was downloaded from DisGeNET [30] including 60,000+ disease-mRNA interactions. We selected 37 diseases common to circRNA-disease interactions and disease-mRNA interactions. There were 820 mRNAs related to these 37 diseases in the initial disease-mRNA interactions. Finally, we constructed a new disease-mRNA association network including 1239 disease-mRNA associations among 37 diseases and 820 mRNAs.

CircRNAs act as miRNA sponges in cells and increase the expression level of target genes. The circRNA-miRNA-mRNA axis plays an important regulatory role in diseases [2,31,32]. In the new circRNA-miRNA associations, we found all the miRNAs related with diseases from circRNA-disease associations or mRNAs from disease-mRNA associations. Furthermore, in the new disease-mRNA associations, we found all the mRNAs related to the 125 of 142 miRNAs mentioned above.

#### 2.1.3. Construction of the Interaction Network

For convenience, we formulate circRNA-disease associations as a binary matrix $Y\in R^{624\times 102}$. If there exists an experimentally verified interaction between circRNA *c_i_* and disease *d_j_*, Y(i,j)=1; otherwise, Y(i,j)=0. At the same time, a circRNA-miRNA interaction matrix and a disease-mRNA interaction matrix are constructed in the same way based on circRNA-miRNA associations and disease-mRNA associations, respectively.

### 2.2. Similarity Calculation

#### 2.2.1. Network Similarity

Zhou et al. [33] demonstrated the usefulness of network similarity. For a given miRNA *mi_k_*, we denote the set of its interacting circRNAs by *C*(*mi_k_*). For a given mRNA *m_k_*, we denote the set of its interacting diseases by *D*(*m_k_*). The network contribution of miRNA *mi_k_* in the circRNA-miRNA interaction network can be calculated as follows

(1) $$nc(mi_{k})=-\ln\left(\left|C(mi_{k})\right|/\sum_{k=1}^{y}\left|C(mi_{k})\right|\right),$$

where *nc*(*mi_k_*) is the network contribution of miRNA *mi_k_* in the circRNA-miRNA interaction network, and *y* is the number of miRNAs. The network contribution of mRNA *m_k_* in the disease-mRNA interaction network can be calculated as follows

(2) $$nc(m_{k})=-\ln\left(\left|D(m_{k})\right|/\sum_{k=1}^{z}\left|D(m_{k})\right|\right),$$

where *nc*(*m_k_*) is the network contribution of mRNA *m_k_* in the disease-mRNA interaction network, and *z* is the number of mRNAs. We also denote the set of miRNAs that interact with a given circRNA *c_u_* by *Mi*(*c_u_*), and the set of mRNAs that interact with a given disease *d_u_* by *M*(*d_u_*). The network similarity between circRNA *c_u_* and circRNA *c_v_* can be defined as

(3) $$CNS(c_{u},c_{v})=\sum_{mi_{k}\in Mi(c_{u})\cap Mi(c_{v})}nc(mi_{k}),$$

where *CNS*(*c_u_*, *c_v_*) is the network similarity between circRNA *c_u_* and circRNA *c_v_*. Similarly, given two diseases, *d_u_* and *d_v_*, the network similarity between disease *d_u_* and disease *d_v_* can be defined as

(4) $$DNS(d_{u},d_{v})=\sum_{m_{k}\in M(d_{u})\cap M(d_{v})}nc(m_{k}),$$

where *DNS*(*d_u_*, *d_v_*) is the network similarity between disease *d_u_* and disease *d_v_*.

#### 2.2.2. Information Entropy Similarity

Information entropy is also used to measure topological similarities of circRNAs and diseases. For a given circRNA *c_u_*, we denote the set of its interacting diseases by $T_{m}^{c_{u}}$. For a given circRNA *c_v_*, we denote the set of its interacting circRNAs by $T_{m}^{c_{v}}$. Next, the information entropy of $T_{m}^{c_{u}}$ can be calculated as

(5) $$\left\{\begin{array}{c} H(T_{m}^{c_{u}})=-\sum_{i=1}^{nd}p(T_{m}^{c_{u}}(i))\log_{2}(p(T_{m}^{c_{u}}(i)))\\ p(T_{m}^{c_{u}}(i))=n(T_{m}^{c_{u}}(i))/N_{cd} \end{array}\right.,$$

where *nd* is the number of diseases related with circRNA *c_u_*, *N_cd_* is the total number of known circRNA-disease interactions, $n(T_{m}^{c_{u}}(i))$ is the number of interactions between the *i*th disease in the related disease set of circRNA *c_u_* and all circRNAs, and $p(T_{m}^{c_{u}}(i))$ is the rate of the *i*th disease in the related disease set of circRNA *c_u_* with the known circRNA-disease interactions. The information entropy similarity between circRNA *c_u_* and circRNA *c_v_* can be calculated as

(6) $$CES(c_{u},c_{v})=\frac{2*H(T_{m}^{c_{u}}\cap T_{m}^{c_{v}})}{H(T_{m}^{c_{u}})+H(T_{m}^{c_{v}})},$$

where $H(T_{m}^{c_{u}}\cap T_{m}^{c_{v}})$ is the information entropy of the intersection of $T_{m}^{c_{u}}$ and $T_{m}^{c_{v}}$, and *CES*(*c_u_*, *c_v_*) is the information entropy similarity of circRNA *c_u_* and circRNA *c_v_*.

Similarly, the information entropy similarity between disease *d_u_* and disease *d_v_* can calculated as follows

(7) $$DES(d_{u},d_{v})=\frac{2*H(T_{n}^{d_{u}}\cap T_{n}^{d_{v}})}{H(T_{n}^{d_{u}})+H(T_{n}^{d_{v}})},$$

where $T_{n}^{d_{u}}$ is the set of disease *d_u_*s interacting circRNAs, $T_{n}^{d_{v}}$ is the set of disease *d_v_*s interacting circRNAs, $H(T_{n}^{d_{u}}\cap T_{n}^{d_{v}})$ is the information entropy of the intersection of $T_{n}^{d_{u}}$ and $T_{n}^{d_{v}}$, and *DES*(*d_u_*, *d_v_*) is the information entropy similarity of disease *d_u_* and disease *d_v_*.

#### 2.2.3. Disease Symptom Similarity

According to the co-occurrence of diseases and symptom terms recorded in the PubMed bibliography, and the work of Zhou et al. [34], the disease similarity can be measured and a symptom-based human disease network can be constructed. Here, the symptom-based disease similarity matrix *DSS* was obtained from the symptom profiles of diseases.

#### 2.2.4. Integration of Similarities

The integrated circRNA similarities and integrated disease similarities are regarded as circRNA features and disease features, respectively. The integrated circRNA similarities can be calculated as follows:

(8) $$ICS(c_{u},c_{v})=\left\{\begin{array}{c} \beta \times CNS(c_{u},c_{v})+(1-\beta )\times CES(c_{u},c_{v})\\ CES(c_{u},c_{v}) \end{array}\right.,$$

where *CNS*(*c_u_*, *c_v_)* is the circRNA network similarity between circRNA *c_u_* and circRNA *c_v_*, *CES*(*c_u_*, *c_v_*) is the circRNA information entropy similarity between circRNA *c_u_* and circRNA *c_v_*, and *ICS*(*c_u_*, *c_v_*) is the integrated circRNA similarity between circRNA *c_u_* and circRNA *c_v_*. β is an adjusting parameter. The dimensions of the *ICS* matrix are 624 × 624.

The integrated disease similarities can be calculated as follows

(9) $$IDS(d_{u},d_{v})=\left\{\begin{array}{c} \alpha \times (DNS(d_{u},d_{v})+DSS(d_{u},d_{v}))+(1-\alpha )\times DES(d_{u},d_{v})\\ DES(d_{u},d_{v}) \end{array}\right.,$$

where *DNS*(*d_u_*, *d_v_*) is the disease network similarity between disease *d_u_* and disease *d_v_*, *DES*(*d_u_*, *d_v_*) is the disease information entropy similarity between disease *d_u_* and disease *d_v_*, *DSS*(*d_u_*, *d_v_*) is the disease symptom similarity between disease *d_u_* and disease *d_v_*, and *IDS*(*d_u_*, *d_v_*) is the integrated disease similarity between disease *d_u_* and disease *d_2_*. α is an adjusting parameter. The dimensions of the *IDS* matrix are 102 × 102.

### 2.3. Graph Attention Network

GAT [35] combines a weighted sum of the adjacent node features with the attention mechanism. The weight of the adjacent node features is completely dependent on the node features and independent of graph structure. GAT aims to construct a hidden self-attention layer and to learn representations for nodes on a graph by assigning different weights to different nodes in a neighborhood.

The input of graph attention layer is

(10) $$f=\{f_{1},f_{2},\cdot \cdot \cdot ,f_{N}\},f_{i}\in R^{F},$$

where *N* is number of nodes (all circRNAs and all diseases), *F* is the length of features, and matrix $f\in R^{N\times F}$ denotes the features of all nodes.

The output of the graph attention layer is

(11) $$f^{\prime }=\{f_{1}^{\prime },f_{2}^{\prime },\cdot \cdot \cdot ,f_{N}^{\prime }\},f_{i}^{\prime }\in R^{F\prime },$$

where $F^{\prime }$ denotes the dimension of new features and matrix $f^{\prime }\in R^{N\times F^{\prime }}$ denotes the new features of all nodes.

The first step is to learn the importance of the neighbors for a given node. GAT implements the self-attention mechanism for every node. The attention coefficient *e_ij_* for an association pair between circRNA *c_i_* and disease *d_j_* is formulated as follows

(12) $$e_{ij}(c_{i},d_{j})=att(Wf_{i},Wf_{j}),$$

where *att* denotes a single-layer feed-forward neural network that transforms input features into high-level features for circRNAs and diseases, and $W\in R^{F^{\prime }\times F}$ is a weight matrix.

To make the attention coefficient comparable across different nodes, GAT further normalizes the attention coefficient *e_ij_* as follows

(13) $$\theta_{ij}=softmax(e_{ij})=\frac{\exp(e_{ij})}{\sum_{t\in N_{i}}\exp(e_{it})},$$

where *N_i_* is the set of neighbor nodes of circRNA *c_i_*, and $\theta_{ij}$ is the normalized attention coefficient indicating the importance of disease *d_j_* for circRNA *c_i_* in the process of information propagation.

By combining Formulas (12) and (13), the complete attention mechanism can be obtained as follows

(14) $$\theta_{ij}=\frac{\exp(leakyReLu(a^{T}[Wf_{i}||Wf_{j}]))}{\sum_{t\in N_{i}}\exp(leakyReLu(a^{T}[Wf_{i}||Wf_{t}]))},$$

where *leakyReLu* is a nonlinearity activation function assigning all negative values a non-zero slope, *T* denotes transposition, || is the concatenation operation, and $a\in R^{2F^{\prime }}$ is the weight coefficient matrix of the graph attention layer.

The second step is to fuse the representations of the neighbors for a given node according to their attention coefficients. The embedding of a given node can be fused by the projected node features of neighbors with different weights as follows

(15) $$f_{i}^{\prime }=\sigma (\sum_{t\in N_{i}}\theta_{it}Wf_{t}),$$

where σ is a nonlinear activation function.

GAT applies a multi-head attention mechanism to increase the stability of the learning process of self-attention. Multi-head attention is the combination of multiple self-attention structures. Each head learns the features in different representation spaces, and the focus of attention learned by multiple heads may be slightly different, which increases the capacity of the model. Specifically, *K*-independent attention mechanisms are integrated to achieve embedding as follows:

(16) $$f_{i}^{\prime }=\sigma (\frac{1}{K}\sum_{k=1}^{K}\sum_{t\in N_{i}}\theta_{it}^{k}\cdot W^{k}f_{t}),$$

where *K* is the number of attention mechanisms and $W^{k}$ is the weight matrix for the *k*th attention mechanism.

Ultimately, the probability score matrix *S* can be calculated as follows

(17) $$S=U\times V^{T},$$

where $U\in R^{nc\times F^{\prime }}$ is the final representation matrix of the circRNAs, in which *nc* is the number of circRNAs; and $V\in R^{nd\times F^{\prime }}$ is the final representation matrix of diseases, in which *nd* is the number of diseases. The dimension of probability score matrix *S* is nc×nd.

**Figure 2:**
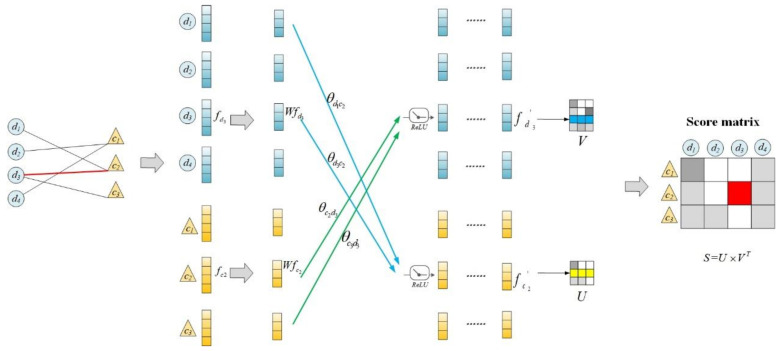
The detailed procedures of using GAT to predict the associations between circRNAs and diseases.

The detailed procedures of using GAT to predict the associations between circRNAs and diseases are shown in Figure 2. As shown in Figure 2, the circRNA-disease association network is fed into a GAT in which the final node representation is obtained through feature propagation and attention fusion. Finally, the prediction score is calculated according to node representation. In the case of disease *d*_3_ and circRNA *c*_2_, the dark blue row represents *d*_3_, the dark yellow row represents *c*_2_, and the red grid represents the predicted score of the association between *d*_3_ and *c*_2_.

## 3. Results

### 3.1. Performance Evaluation

The five-fold cross-validation (5CV) technique was used to evaluate the prediction performance of our model. The 5CV technique randomly divides the positive samples into five equal parts, and takes out one part of them as a testing sample while the rest of samples are regarded as training samples. Next, the predicted scores are sorted in descending order. We drew the receiver operating characteristics (ROC) curve via plotting the true positive rate (TPR) versus the false positive rate (FPR) at different score thresholds. TPR (FPR) refers to the percentage of positive (negative) cases that are correctly identified. Generally, the area under the ROC curve (AUC) is calculated and employed to evaluate the prediction performance. Specifically, the closer the AUC value is to one, the better the prediction performance. As a result, in 5CV, GATCDA achieved an AUC of 0.9011. In addition, GATCDA yielded an accuracy of 0.8710, with a precision of 0.9013.

### 3.2. Adjustment of Parameters

**Figure 3:**
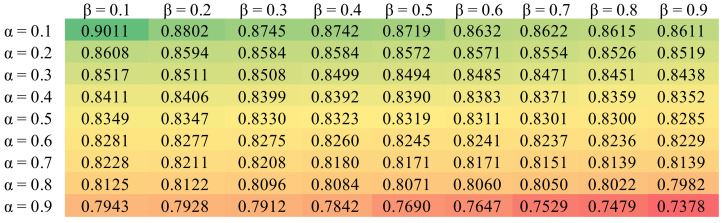
Heatmap of AUC results of adjustment parameters.

The GATCDA model involves two parameters, α and β, which adjust the influence of similarity data when calculating integrated similarities. We let α and β both range between 0.1 and 0.9. As a result, GATCDA (α = 0.1, β = 0.1) gained the highest AUCs of 0.9011 in 5CV as shown in Figure 3.

### 3.3. Compared with Other Methods

**Figure 4:**
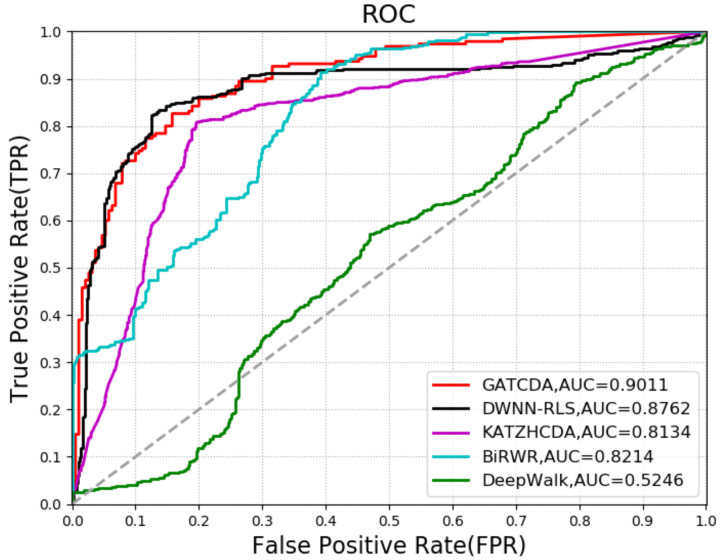
The ROC curves and AUCs of five methods using 5CV.

**Figure 5:**
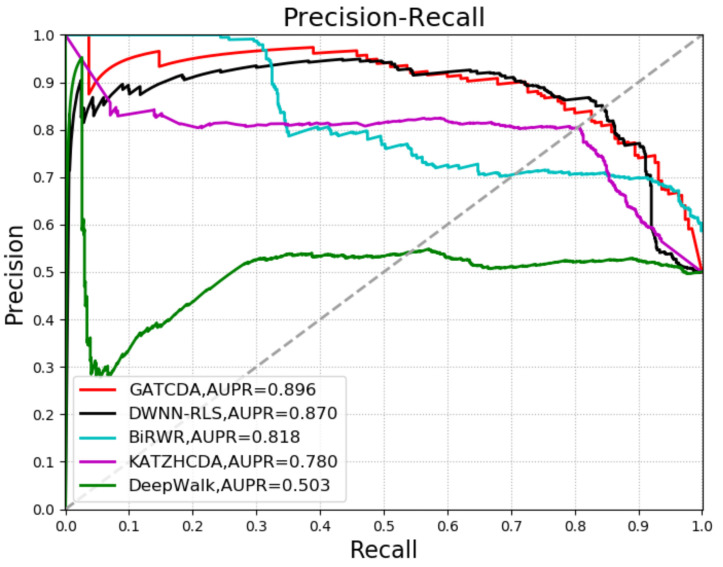
Comparison of five methods in PR curves and AUPRs (5CV).

To analyze the performance of GATCDA in predicting circRNA-disease associations, we compared GATCDA with other four methods: DWNN-RLS [36], KATZHCDA [21], bi-random walks (BiRWR) [37], and DeepWalk [38]. DWNN-RLS uses the regularized least squares of the Kronecker product kernel to predict circRNA-disease associations. KATZHCDA uses KATZ measures for human circRNA-disease association prediction. BiRWR predicts circRNA-disease associations by walking in a circRNA subnetwork and disease subnetwork. DeepWalk is a way to learn the potential representation of nodes in a graph structure. The ROC curve and AUC value of each method using 5CV are shown in Figure 4. The precision-recall curve and the area under the precision-recall curves (AUPR) value of each method with 5CV are shown in Figure 5. Through comparison, it can be seen that the extraction of circRNA and disease features by GAT can achieve better prediction performance compared with DeepWalk. In addition, as a deep learning method, GAT also shows better prediction performance compared with the other two link-based prediction methods (KATZHCDA and BiRWR).

### 3.4. Case Study

To further evaluate the prediction performance of GATCDA, we also carried out case studies on three common diseases, i.e., bladder cancer, diabetes retinopathy, and rheumatoid arthritis.

Bladder cancer is the most frequent cancer affecting the urinary tract [39], and has a high rate of recurrence [40]. Diabetes retinopathy is a common chronic metabolic disorder, increasing with an ageing population and the growing number of cases of diabetes [41]. Rheumatoid arthritis is the most common chronic inflammatory arthritis, which can lead to cartilage and bone damage and disability [42]. There is increasing evidence that circRNAs can be used as effective biomarkers for the diagnosis of bladder cancer, diabetes retinopathy, and rheumatoid arthritis. Therefore, we selected bladder cancer, diabetes retinopathy, and rheumatoid arthritis to verify the predictive ability of GATCDA.

In this work, all known associations between the investigated disease and circRNAs were assumed to be unknown. Through the calculation of GATCDA, the circRNAs with the top 10 scores were selected among all the predicted associations between the investigated disease and circRNAs. Then, through searching the related literature, some circRNAs were confirmed to be related to the investigated disease. 

The results of the case studies of the three diseases (bladder cancer, diabetes retinopathy, and rheumatoid arthritis) are shown in Table 1. For bladder cancer, we can see that 8 of the top 10 candidates with the highest prediction scores are confirmed by the relevant literature. Notably, the seventh circRNA (hsa_circ_0075828) predicted by GATCDA is related to bladder cancer. For diabetes retinopathy and rheumatoid arthritis, 7 of the top 10 candidates with the highest prediction scores are confirmed by the relevant literature. For example, Li et al. found that hsa_circ_0001859 regulates ATF2 expression by functioning as an MiR-204/211 sponge in human rheumatoid arthritis [43]. Zhang et al. revealed that hsa_circ_0005015 acts as an miR-519d-3p sponge to inhibit miR-519d-3p activity, leading to increasing MMP-2, XIAP, and STAT3 expression in diabetes retinopathy [44].

In order to verify the prediction performance of GATCDA, we compared it with other models in the case studies of these three kinds of same diseases, as shown in Table 2. From Table 2, 8, 7, and 7 out of these top 10 circRNAs predicted by GATCDA were verified to be associated with bladder cancer, diabetes retinopathy, and rheumatoid arthritis, respectively, which are the highest among competing methods. Therefore, GATCDA also outperforms the competing prediction models in terms of the hit rate in case studies.

## 4. Discussion

With the rapid development in RNA sequencing technology and bioinformatics analysis, various studies have shown that circRNAs are closely related to the occurrence and development of disease, and circRNAs act a potential biomarkers for patients with certain cancers. Therefore, discovering associations between circRNAs and diseases is significative for disease diagnosis and treatment. Nevertheless, biological experiments are very costly in terms of time and money. It has become a hot topic to predict associations between circRNAs and diseases using computational methods in recent years. The lack of data on the interactions between circRNA and disease limits the predictive power of most computational methods.

In this study, we proposed a new computational model named GATCDA to identify underlying circRNA-disease associations. We performed 5CV experiments to assess the predictive performance of GATCDA. Our method yielded an AUC value of 0.9011 and an AUPR value of 0.896, which are higher than those of DWNN-RLS, KATZHCDA, BiRWR, and DeepWalk. In addition, the predicted top 10 circRNA-disease interactions in the case studies of three diseases (bladder cancer, diabetes retinopathy, and rheumatoid arthritis) have been confirmed in the relevant literature, which suggests that GATCDA can be an effective tool for predicting circRNA-disease associations.

The accurate predictive performance of GATCDA is attributed to the following factors: First, in order to identify more interactions between circRNAs and diseases, circRNA-disease interactions were integrated from four databases, i.e., CircR2Disease, CircAtlas 2.0, Circ2Disease, and CircRNADisease. Therefore, the number of positive samples input to GAT algorithm is higher. Second, as the circRNA-miRNA-mRNA axis plays an important role in the generation and development of diseases, the circRNA-miRNA interactions and disease-mRNA interactions were adopted to construct features, in which mRNAs are related to 88% of miRNAs. CircRNAs have several distinct modes of action. From the functional perspective of circRNAs as miRNA sponges, an interaction network can be constructed for circRNA network similarity calculations. Other functions of circRNA are difficult to quantify. Third, more similarities are involved in GATCDA, i.e., disease symptom similarity, disease network similarity, disease information entropy similarity, circRNA network, and circRNA information entropy similarity, which are integrated effectively. Fourth, GAT has obvious advantages, including learning representations for nodes on a graph using an attention mechanism. Therefore, it can assign different weights to different nodes in a neighborhood.

GATCDA also has limitations. Compared with other non-coding RNAs, the interactions between circRNAs and diseases are still insufficient. Therefore, the circRNA-disease association matrix is sparse, which has an impact on prediction performance. In the future, we will collect more data on the associations between circRNAs and diseases.

## 5. Conclusions

In this study, we proposed a new computational model named GATCDA to identify underlying circRNA-disease associations. Specifically, GAT was used to predict circRNA-disease associations based on multiple similarities of circRNA and disease. This work has two highlights: First, as the circRNA-miRNA-mRNA axis plays an important role in the generation and development of diseases, circRNA-miRNA interactions and disease-mRNA interactions are adopted to construct features, in which mRNAs are related to 88% of miRNAs. Second, GAT is used to predict the interactions between circRNAs and diseases. GAT can assign different learning weights to different neighbors, and the correlation between vertex features can be better integrated into the model. In terms of predictive performance, GATCDA achieves an AUC of 0.9011 in 5CV, and in case studies of three diseases, 70% of experimentally validated relationships were predicted. In summary, GATCDA is a powerful tool for predicting circRNA-disease associations.

## Figures and Tables

**Table 1:** Candidate circRNAs identified by GATCDA for bladder cancer, diabetes retinopathy and rheumatoid arthritis.

Disease	Rank	CircRNA	Source
Bladder cancer	1	hsa_circ_0091017	[45]
	2	hsa_circ_0002495	[46]
	3	hsa_circ_0071410	- ^1^
	4	hsa_circ_0001141	[47]
	5	hsa_circ_0007915	-
	6	hsa_circ_0041103	[48]
	7	hsa_circ_0075828	[49]
	8	hsa_circ_0061265	[48]
	9	hsa_circ_0002768	[50]
	10	hsa_circ_0082582	[48]
Diabetes retinopathy	1	hsa_circ_0098964	-
	2	hsa_circ_0057093	[44]
	3	hsa_circ_0051172	-
	4	hsa_circ_0087215	[44]
	5	hsa_circ_0081162	[44]
	6	hsa_circ_0066922	[44]
	7	hsa_circ_0026388	[44]
	8	hsa_circ_0005525	-
	9	hsa_circ_0000615	[51]
	10	hsa_circ_0005015	[44]
Rheumatoid arthritis	1	hsa_circ_0083964	[52]
	2	hsa_circ_0064996	[52]
	3	hsa_circ_0004712	[52]
	4	hsa_circ_0061893	-
	5	hsa_circ_0052012	[52]
	6	hsa_circ_0032683	[52]
	7	hsa_circ_0001859	[43]
	8	hsa_circ_0088036	[53]
	9	hsa_circ_0003028	-
	10	hsa_circ_0010090	-

^1^—means has no source.

**Table 2:** The number of circRNAs confirmed by evidence in the top 10 potential disease-related circRNAs predicted by GATCDA and other models in case studies of the three kinds of diseases such as bladder cancer, diabetes retinopathy, and rheumatoid arthritis.

Model	Bladder Cancer	Diabetes Retinopathy	Rheumatoid Arthritis
GATCDA	8	7	7
DWNN-RLS	7	5	4
KATZHCDA	5	4	4
BiRWR	5	3	4
DeepWalk	3	1	2

## Data Availability

The data presented in this study are available on request from the corresponding authors.
